# Spinocerebellar ataxias: from pathogenesis to recent therapeutic advances

**DOI:** 10.3389/fnins.2024.1422442

**Published:** 2024-06-04

**Authors:** Zi-Ting Cui, Zong-Tao Mao, Rong Yang, Jia-Jia Li, Shan-Shan Jia, Jian-Li Zhao, Fang-Tian Zhong, Peng Yu, Ming Dong

**Affiliations:** ^1^Department of Neurology and Neuroscience Center, The First Hospital of Jilin University, Changchun, China; ^2^Department of Plastic and Reconstructive Surgery, The First Hospital of Jilin University, Changchun, China; ^3^Department of Ophthalmology, the Second Hospital of Jilin University, Changchun, China

**Keywords:** spinocerebellar ataxias, gene therapy, disease-modifying molecular therapies, neurodegenerative disorders, RNA interference, polyQ diseases

## Abstract

Spinocerebellar ataxia is a phenotypically and genetically heterogeneous group of autosomal dominant-inherited degenerative disorders. The gene mutation spectrum includes dynamic expansions, point mutations, duplications, insertions, and deletions of varying lengths. Dynamic expansion is the most common form of mutation. Mutations often result in indistinguishable clinical phenotypes, thus requiring validation using multiple genetic testing techniques. Depending on the type of mutation, the pathogenesis may involve proteotoxicity, RNA toxicity, or protein loss-of-function. All of which may disrupt a range of cellular processes, such as impaired protein quality control pathways, ion channel dysfunction, mitochondrial dysfunction, transcriptional dysregulation, DNA damage, loss of nuclear integrity, and ultimately, impairment of neuronal function and integrity which causes diseases. Many disease-modifying therapies, such as gene editing technology, RNA interference, antisense oligonucleotides, stem cell technology, and pharmacological therapies are currently under clinical trials. However, the development of curative approaches for genetic diseases remains a global challenge, beset by technical, ethical, and other challenges. Therefore, the study of the pathogenesis of spinocerebellar ataxia is of great importance for the sustained development of disease-modifying molecular therapies.

## 1 Introduction

Hereditary cerebellar ataxia encompasses a highly heterogeneous group of neurodegenerative disorders that are major causes of cerebellar ataxia, with modes of inheritance involving autosomal dominant, autosomal recessive, X-linked, or mitochondrial inheritance. Autosomal dominant cerebellar ataxia, also known as spinocerebellar ataxia (SCA), involves a wide variety of causative genes and mutation types, and in most cases lacks a clear genotype–phenotype correlation, which makes it difficult to reliably differentiate between the SCA subtypes by the clinical phenotype. Therefore, genetic testing is the primary diagnostic tool for this condition. There have been tremendous advances in this field owing to the emergence and application of genetic testing technologies, and the list of genes associated with SCA has been continuously expanding in recent years (Chen et al., 2015). The clinical features of SCAs overlap with each other, and their core symptoms typically consist of gait instability, ataxia, nystagmus, and dysarthria. Some SCAs present as pure cerebellar syndromes, whereas the majority are frequently accompanied by nonataxic symptoms. The treatment approaches for SCAs are currently limited to symptomatic therapy. Owing to the commonalities in pathogenesis, developmental strategies for disease-modifying therapies applicable to multiple subtypes have been proposed. However, there are different causative proteins of each SCA subtype, and the search for broadly applicable therapeutic approaches should be accompanied by continued research into the pathogenesis and treatment of each disease. The purpose of this review was to broaden our understanding of the status of SCA research by presenting common clinical entities, describing their pathogenesis, and discussing the current and future therapeutic perspectives.

## 2 Pathogenic mechanisms

**Figure 1:**
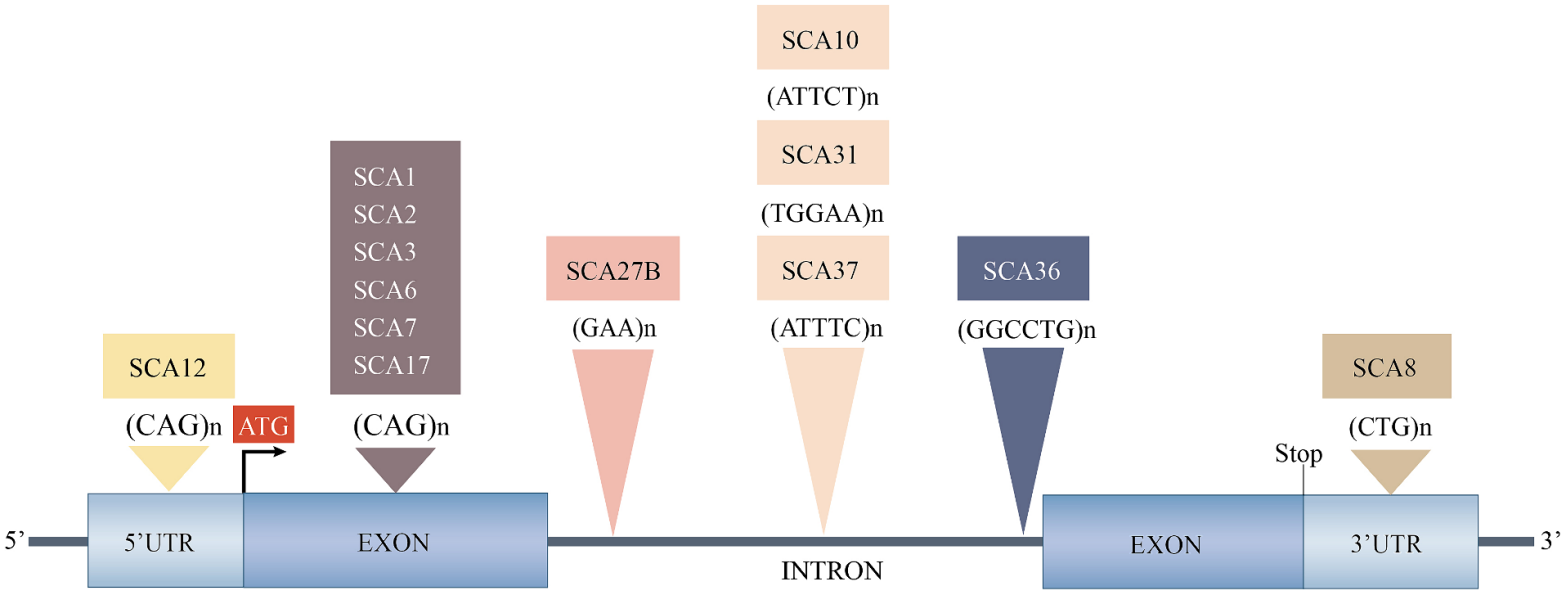
Spinocerebellar ataxias caused by expanded repeats. 3’UTR, 3′ untranslated region; 5’UTR, 5′ untranslated region; SCA, spinocerebellar ataxia. Reproduced from Ashizawa et al. (2018) with permission from Springer Nature.

Based on the genetic nomenclature, the 50 identified SCAs are numbered in accordance with the order of discovery of their genetic locus. Moreover, SCAs are categorized into two groups according to their underlying mutation type into repeat expansions and point mutations. The former can be further categorized into SCAs induced by polyglutamine (polyQ)-encoding CAG repeat expansions and noncoding repeat expansions, owing to the different pathogeneses of the diseases. Among the SCAs induced by repeat expansion, six were due to the expanded CAG repeats encoding polyQ in their respective genes, which is the most common type. Since other neurodegenerative diseases, such as Huntington’s disease (HD) and dentatorubral-pallidoluysian atrophy (DRPLA), share the same mechanism, they are collectively referred to as polyQ diseases (Durr, 2010). Some expanded repeats that cause SCAs are translated into different frames by a mechanism independent of the start codon (AUG) called repeat-associated non-AUG translation (RAN). This mechanism may also act on the antisense transcripts of the opposite strand. For example, in SCA8, noncoding CTG repeat expansion is transcribed from the 3′ untranslated region (3’UTR) of the *ATXN8OS* gene. Another gene, *ATXN8*, on the opposite strand, can generate an antisense transcript, and its CAG repeats can produce polyQ-containing proteins through RAN translation. Expanded CAG repeats in SCA12 occur in the 5′ untranslated region (5’UTR) of the *PPP2R2B* gene. Additionally, SCA10, SCA31, SCA36, and SCA37 are caused by expanded repeats in the intronic region. A heterozygous point mutation in FGF14 is the underlying cause of SCA27A. Recently, heterozygous GAA repeat expansion in intron 1 of FGF14 was reported to contribute to late-onset ataxia, referred to as SCA27B (Figure 1 and Table 1) (Coarelli et al., 2023a). Most SCAs caused by point mutations are results of missense mutations, whereas a small proportion are caused by deletions, translocations, or duplications of small DNA fragments (Table 2) (Ashizawa et al., 2018).

**Table 1:** Spinocerebellar ataxias caused by repeat expansions.

Disease	Gene and protein (repeat location)	Repeats, principal repeat unit			Notable characteristic clinical signs
		Normal	Intermediate	Disease	
**SCAs caused by polyglutamine-coding CAG repeat expansions**					
SCA1	*ATXN1*	6–39	40	41–83	Hypermetric saccades, pyramidal signs
SCA2	*ATXN2*	<31	31–33	34–200	Slow saccades, areflexia
SCA3	*ATXN3*	12–44	45–55	56–86	Bulged eyes, motor neuron signs
SCA6	*CACNA1A*	<18	19	20–33	Downbeat nystagmus
SCA7	*ATXN7*	4–19	28–33	34- > 460	Visual loss
SCA17	*TBP*	25–40	-	41–66	Huntington disease-like
**SCAs caused by noncoding repeat expansions**					
SCA8	*OSATXN8* (3’UTR)	15–34, CTG or CAG	34–89, CTG or CAG	89–250, CTG or CAG	Reduced penetrance
SCA10	*ATXN10* (intron)	8–32 ATTCT	33–799 ATTCT	800–4,500, ATTCT	Some families with epilepsy
SCA12	*PPP2R2B* (5’UTR)	7–28, CAG	29–66, CAG	67–78, CAG	Tremor
SCA27B*	*FGF14* (intron)	Unknown	Unknown	250–300 or > 300, GAA	Late-onset episodic ataxia
SCA31	*BEAN* (intron)	<400, ATTTT	Unknown	500–760, TGGAA	Pure cerebellar ataxia
SCA36	*NOP56* (intron)	3–14, GGCCTG	Unknown	650–2,500	Motor neuron disease
SCA37	*DAB1* (5’UTR; intron)	<400, ATTTT	Unknown	31–75, ATTTC	Pure cerebellar ataxia

SCA, spinocerebellar ataxia.

SCA27B*: Repeats between 250 and 300 were pathogenic but showed incomplete penetrance, while repeats >300 showed complete penetrance.

Data were extracted from OMIM and GeneReviews of corresponding SCAs.

Reproduced from Ashizawa et al. (2018) with permission from Springer Nature.

**Table 2:** Spinocerebellar ataxias caused by point mutations.

Disease	Gene and protein	Mutation	Notable characteristic clinical signs
SCA5	*SPTBN2*	Missense, in-frame deletion	Downbeat nystagmus and some patients with spasticity, anticipation
SCA11	*TTBK2*	Frameshift	Some patients with pyramidal signs
SCA13	*KCNC3*	Missense	Variable between families
SCA14	*PRKCG*	Missense or exon deletions	Tremor or myoclonus, facial myokymia
SCA15 and SCA16	*ITPR1*	Exon deletions	Pure cerebellar ataxia with tremor
SCA18	*IFRD1*	Missense	Sensorimotor neuropathy
SCA19 and SCA22	*KCND3*	Missense or in-frame deletions	Extracerebellar features variable between families
SCA20	*Multiple*	260 kb duplication	Pure cerebellar ataxia with spasmodic dysphonia, palatal tremor
SCA21	*TMEM240*	Missense	Cognitive impairment, extrapyramidal signs
SCA23	*PDYN*	Missense	Extracerebellar features variable between families
SCA25	*PNPT1*	Splice site	Sensory neuropathy
SCA26	*EEF2*	Missense	Pure cerebellar ataxia
SCA27A	*FGF14*	Missense or frameshift	Mental retardation, tremor
SCA28	*AFG3L2*	Missense or frameshift	Spastic ataxia
SCA29	*ITPR1*	Missense	Pure cerebellar ataxia, congenital nonprogressive
SCA34	*ELOVL4*	Missense	Hyperkeratosis, MSA-C like
SCA35	*TGM6*	Missense or in-frame deletions	Hyperreflexia and variable other extracerebellar features
SCA38	*ELOVL5*	Missense	Pure cerebellar ataxia, some patients have sensory neuropathy
SCA40	*CCDC88C*	Missense	Spastic ataxia
SCA41	*TRPC3*	Missense	Pure cerebellar ataxia
SCA42	*CACNA1G*	Missense	Dementia
SCA43	*MME*	Missense	Peripheral neuropathy
SCA44	*GRM1*	Missense or frameshift	Spasticity
SCA45	*FAT2*	Missense	Pure cerebellar ataxia (single family)
SCA46	*PLD3*	Missense	Sensory neuropathy
SCA47	*PUM1*	Missense	Pure cerebellar ataxia in adults. Juvenile forms have developmental complex phenotype
SCA48	*STUB1*	Missense or frameshift	Cerebellar ataxia or cognitive/affective disorder
SCA49	*SAMD9L*	Missense	Cerebellar ataxia, cytopenia and myeloid malignancies
SCA50	*NPTX1*	Missense	Downbeat nystagmus, myoclonus, cognitive impairment

MSA-C, Multiple system atrophy-cerebellar type; SCA, spinocerebellar ataxia.

Data were extracted from OMIM and GeneReviews of corresponding SCAs.

Reproduced from Ashizawa et al. (2018) with permission from Springer Nature.

### 2.1 The polyQ SCAs

**Figure 2:**
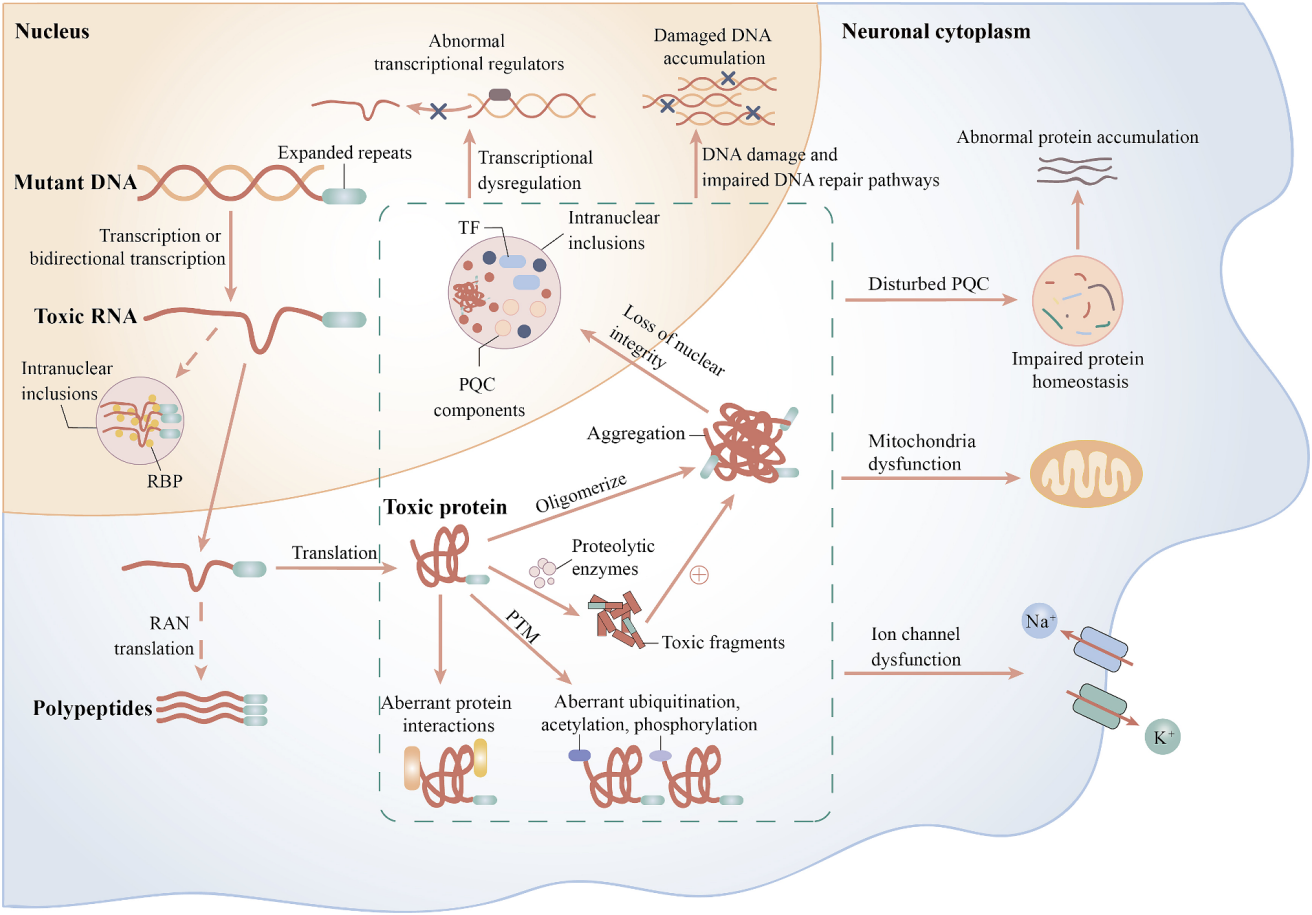
Possible pathogenic mechanisms of spinocerebellar ataxias. Solid arrows indicate possible mechanisms for polyQ SCAs and dashed arrows indicate possible mechanisms for SCAs caused by noncoding repeat expansions. RAN, non-AUG translation; RBP, RNA-binding protein; PQC, protein quality control; PTM, post-translational modifier; TF, transcription factor.

The primary pathogenic mechanism of polyQ SCAs are the toxic effects of the proteins encoded by mutant genes containing expanded CAG repeats, and to a lesser extent, the possible toxic effects of RNA transcripts from mutant genes (Coarelli et al., 2018). Mutated genes can also generate antisense transcripts containing complementary RNA repeat expansions from opposite DNA strands, and bidirectional expression of such expanded repeats occurs in SCA2, SCA7, and SCA8 (Ikeda et al., 2008; Sopher et al., 2011; Li et al., 2016). Furthermore, the disruption of natural protein function is correlated with toxic gain-of-function of aberrant polyQ. Altered proteins tend to form abnormal conformations that alter the way they bind to normal molecular chaperones and readily oligomerize and form intraneuronal oligomers and intranuclear inclusions. These aggregates may be directly toxic or produce toxic effects by sequestering proteins or cellular components, such as transcription factors, molecular chaperones, and components of the cellular clearance machinery, thereby triggering aberrant pathological processes leading to neuronal damage, such as impaired protein quality control (PQC) pathways, protein hydrolytic cleavage, transcriptional and post-translational dysregulation, aberrant protein interactions, ion channel dysfunction, impaired DNA repair, and mitochondrial dysfunction (Figure 2) (Paulson et al., 2017).

The PQC pathways include a ubiquitin-proteasome system, molecular chaperones, and autophagy, and the different components are tightly linked and interact with each other to maintain protein homeostasis and degrade misfolded proteins. Autophagy is involved in the degradation of damaged organelles or toxic protein aggregates, and there is growing evidence suggesting that impaired autophagy is associated with many neurodegenerative diseases such as SCA2, SCA3, Parkinson’s disease, Alzheimer’s disease, and HD (Menzies et al., 2017). The levels of the autophagy marker proteins SQSTM1 and LC3B were altered in SCA2 neuronal cells and lentiviral mouse models, and abnormal accumulation of SQSTM1 and LC3B was observed in the cerebellum and striatum of patients with SCA2, indicating that impaired autophagy may play an important role in the pathogenesis of SCA2 (Marcelo et al., 2021). ATXN3 is directly involved in PQC as a deubiquitinating enzyme, and PQC perturbation in SCA3 may result from a combination of accumulation of mutant ATXN3 aggregates, sequestration of the ubiquitin-proteasome system and autophagy regulators, and destabilization of Beclin1, a key protein of the autophagy pathway (McLoughlin et al., 2020). Almost all polyQ SCAs exhibit extensive mislocalization and aggregation of pathogenic proteins; therefore, the entire type may be associated with disturbed protein homeostasis.

It has been hypothesized that the cleavage of mutant polyQ proteins by proteolytic enzymes may produce more toxic short protein fragments, that are associated with the subsequent induction of aggregate formation. These fragments may translocate to the nucleus and interfere with transcription. Findings from several polyQ disease models, such as SCA3, SCA6, SCA7, and HD, provide evidence for this hypothesis (Buijsen et al., 2019). ATXN3 has multiple proteolytic sites. A study using L-glutamate to excite induced pluripotent stem cell (iPSC)-derived neurons from patients with SCA3 reported that the excitation initiated an inward flow of Ca^2+^, induced intraneuronal cleavage of ATXN3, and led to the formation of insoluble aggregates. The insoluble substances could completely disappear after treatment with a calpain inhibitor, elucidating the critical role of calpain-mediated ATXN3 cleavage in the formation of aggregates (Koch et al., 2011). Therefore, the reduction of mutant protein aggregation may be achieved by inhibiting proteolytic cleavage, such as proteolytic enzyme inhibitors or gene therapy-based removal of protein regions containing proteolytic cleavage sites associated with toxic fragments.

### 2.2 SCAs caused by noncoding repeat expansions

In general, SCA10, SCA31, SCA36, and SCA37 are caused by pentanucleotide or hexanucleotide expansions within nonprotein-coding introns, resulting in the accumulation of toxic nonprotein-coding RNAs containing large expanded repeats in the intranuclear RNA foci. These foci sequester key RNA-binding proteins that perturb RNA-dependent RNA homeostasis, which may lead to cytotoxicity (Figure 2) (Zhang and Ashizawa, 2017). For example, RNA foci formed by the accumulation of AUUCU expansion in SCA10 can sequester heterogeneous cytosolic ribonucleoprotein K and impair its function, and its down-regulation can induce translocation and accumulation of protein kinase Cδ in the mitochondria, which subsequently triggers the caspase-3-mediated apoptotic pathway (Kurosaki and Ashizawa, 2022). Repeated ATTTC expansion of the *DAB1* gene leads to overexpression of DAB1 and induces an RNA switch, causing upregulation of the reelin-DAB1 and PI3K/AKT signaling pathways in the cerebellum of patients with SCA37, leading to cerebellar neuronal degeneration (Corral-Juan et al., 2018).

Bidirectional expression and RAN translation potentially cause the dual pathogenesis of SCA8, with RAN translation products accumulating in cells as toxic aggregates that disrupt the function of nuclear pores and integrity of membrane-free organelles (Figure 2). However, the degree of pathogenicity of RAN translation in relation to the toxic RNA transcripts produced by bidirectional expression remains to be investigated (Lee et al., 2017). In SCA12, expanded CAG repeats in the 5’UTR of *PPP2R2B* have been hypothesized to cause the disease by misregulating the host gene expression through alteration of promoter activity, splicing, and transcript stability. In a recent study, bidirectional expression of repeat sequences, toxic foci of antisense transcripts, and RAN translation were observed in SCA12-iPSCs, iPSC-derived NGN2 neurons, and mouse brains. Therefore, SCA12 may be involved in a pathogenic mechanism similar to that of SCA8 (Cohen and Margolis, 2016; Zhou et al., 2023). SCA27B iPSC-derived motoneurons and cerebellar autopsy specimens of patients with SCA27B showed reduced expression of FGF14 RNA and protein; thus, the causative factor was considered to be a loss of protein function due to transcriptional interference; however, the mechanism leading to FGF14 transcriptional defects still requires further investigation (Rafehi et al., 2023).

### 2.3 SCAs caused by point mutations

The application of NGS in the diagnosis of genetic ataxia has led to a rapidly increasing number of genetic variations, including disease-causing mutations and variations of unknown significance (VUS). Most known disease-causing mutations are missense mutations, and the pathogenic mechanism may involve a toxic gain-of-function of mutant proteins or a dominant negative effect (Klockgether et al., 2019). For example, SCA35 is caused by mutations in the TGM6 gene encoding TG6, which causes neuronal dysfunction and death, including reduced distribution of mutant proteins in the nucleus compared to the baseline levels, loss of enzyme activity, and abnormal accumulation of mutants in the perinuclear region, with reduced solubility and ease of aggregation, leading to the acquisition of toxicity (Tripathy et al., 2017). In addition, haploinsufficiency caused by loss-of-function mutations in genes may also cause SCAs such as SCA15/16, SCA27, and SCA47 (Iwaki et al., 2008; Misceo et al., 2009; Gennarino et al., 2018).

### 2.4 Possible downstream mechanisms

#### 2.4.1 Impaired ion channels

Evidence suggests that impaired ion channels in the cerebellar circuitry result in abnormalities in Purkinje cell firing and alterations in the signaling pathways, contributing to neuronal dysfunction. Two pathways can cause ion channel dysfunction; first, mutations in genes encoding the ion channels themselves, such as SCA6, SCA13, SCA19/SCA22, SCA15/SCA16, SCA29, SCA41, SCA42, and SCA44; and second, mutations in genes encoding proteins that regulate the activity of ion channels, which indirectly cause changes in channel function or expression, mainly polyQ SCAs (Bushart and Shakkottai, 2019).

#### 2.4.2 Mitochondrial dysfunction and impaired mitophagy

Any neurodegenerative disease may involve mitochondrial dysfunction in a common pathway, and SCAs disease proteins may directly or indirectly impair mitochondrial function, resulting in increased oxidative stress and impaired bioenergetics. SCA28 is caused by a missense mutation in *AFG3L2*, which encodes a key component of the mitochondrial m-AAA protease, and a study found that fibroblasts from mutant mice exhibited impaired mitochondrial bioenergetics, reduced mitochondrial membrane potential, and altered mitochondrial network connectivity and morphology, proposing that the slow accumulation of toxic mitochondrial proteins is a pathogenic trigger event (Mancini et al., 2019). One study observed increased mitochondrial oxidative stress, altered mitochondrial respiratory chain enzymes, and mitochondrial morphology in fibroblasts from a patient with SCA2 who had not yet demonstrated clinical symptoms, and another study reported that individuals carrying mutant ATXN3 had a significant loss of mitochondrial DNA content in the preclinical phase. These findings suggest that mitochondrial dysfunction may precede the disease onset (Cornelius et al., 2017; Raposo et al., 2019).

In order to maintain an intact mitochondrial network and thus adequate cellular homeostasis, dysfunctional mitochondria can be selectively removed by the mitophagy process. In neurons, when damaged mitochondria cannot be removed by mitophagy, reactive oxygen species, nitrogen oxides, and other oxidizing substances are produced, which can cause a variety of neurodegenerative diseases, such as Alzheimer’s disease, Parkinson’s disease, and HD (Okatsu et al., 2013). The pathogenic mechanisms of SCA3 and SCA6 have recently been reported to also involve impaired mitophagy, with fibroblasts from SCA3 patients exhibiting altered mitochondrial morphology, impaired bioenergetics, and dysregulation of Parkin-VDAC1-mediated mitophagy processes, and the accumulation of mitochondrial structural damage in SCA6 Purkinje cells has been proposed to may be partially related to impaired mitophagy during late stages of the disease (Wiatr et al., 2021; Harmuth et al., 2022; Leung et al., 2024). The mechanisms of mitophagy discovered so far are classified into ubiquitin-dependent and ubiquitin-independent pathways, and PINK1 and Parkin are key proteins with interactions in the ubiquitin-dependent pathway. PINK1 is a serine/threonine kinase located in depolarized mitochondria, in depolarized, damaged mitochondria, autophosphorylated PINK1 accumulates in the outer mitochondrial membrane and recruits Parkin located in the cytoplasm (Lu et al., 2023). One known substrate of ataxin-3 is Parkin, which acts as an E3 ubiquitin-protein ligase that ubiquitinates mitochondrial outer membrane proteins, thereby recruiting autophagy proteins to induce mitochondrial degradation. A recent report proposed that in SCA3 disease, mutant ATXN3 is associated with an aberrant loss of Parkin, leading to a reduction in VDAC1 polyubiquitination, which impedes mitophagy and initiates the apoptotic pathway through multiple programs, and ultimately an apoptotic process that leads to cell death and neurodegeneration (Harmuth et al., 2022). Further studies are needed in the future to validate the role of mutant ATXN3 in causing dysregulation of the PINK1/Parkin pathway, to provide a theoretical basis for expanding the understanding of the pathogenesis of SCA3, discovering new therapeutic targets, and designing therapeutic approaches targeting the mitophagy process. Thus, the use of modulators of PINK1/Parkin pathway activity may be able to promote the onset of mitophagy and improve mitochondrial quality control. Previous studies have identified a number of pharmacological molecules with such effects, such as triphosphate kinetin, which has a higher affinity for PINK1 than the natural substrate ATP, and whose application to cells may promote Parkin recruitment levels in depolarized mitochondria by enhancing PINK1 activity. There are also studies focusing on Parkin, the tumor suppressor p53 may inhibit mitophagy by preventing its translocation to mitochondria through direct interaction with Parkin, and p53 inhibitor may improve mitophagy through a feedback loop between Parkin and p53 (Zhang et al., 2011; Osgerby et al., 2017; Zhang et al., 2020).

#### 2.4.3 Transcriptional dysregulation

The pathogenesis of SCAs may involve multiple molecular mechanisms that lead to transcriptional dysregulation, such as protein-DNA interactions, acetylation, phosphorylation, and RNA interference. ATXN1 is a transcriptional cofactor that interacts with transcriptional regulators, RNA splicing factors, and some nuclear receptors during gene expression, and SCA1 pathogenesis is associated with altered interactions between ATXN1 and other proteins (Srinivasan and Shakkottai, 2019). Expanded ATXN1 facilitates the formation of transcriptional repressor complexes by binding to homologs of the transcriptional repressor protein capicua. Studies on mouse models and iPSCs from patients with SCA1 have shown that the ATXN1-capicua complex drives cerebellar toxicity through a gain-of-function mechanism (Rousseaux et al., 2018). The PolyQ expansion in SCA17 occurs within the transcription factor TATA box-binding protein (TBP), and impaired transcriptional activity caused by mutating TBP may be the main form of toxicity that leads to the pathogenesis of SCA17 (Yang et al., 2016). Furthermore, SCA47 is caused by mutations in the *PUM1* gene which belongs to a family of RNA-binding proteins that regulate mRNA stability and inhibit translation. Experiments on patient fibroblasts showed that *PUM1* missense mutations reduced PUM1 protein levels to varying degrees, whereas the levels of PUM1 targets, including ATXN1 and E2F3, were increased. PUM1-deficient mouse models exhibited progressive Purkinje cell degeneration and loss of dendritic arborization, producing a phenotype similar to that of SCA1 mice (Gennarino et al., 2015, 2018). ATXN7 is a component of the SAGA histone acetyltransferase complex and polyQ expansion disrupts its structural integrity, leading to transcriptional dysregulation, abnormal chromatin acetylation, and altered gene expression (McCullough and Grant, 2010).

#### 2.4.4 Other possible mechanisms

DNA damage and impaired DNA repair pathways may also be involved in the pathogenesis of SCAs. Wild-type ATXN3 and its interacting proteins play a role in DNA repair factor recruitment, cell cycle arrest, DNA repair, and accumulation of DNA damage in the SCA3 animal model (Chatterjee et al., 2015; McLoughlin et al., 2020). Mutant PolyQ SCA proteins are frequently concentrated in the nucleus and can form intranuclear inclusions, indicating disruption of transmembrane transport and loss of nuclear integrity. Each SCA subtype may have different downstream consequences owing to differences in the structure, function, and location of normal protein expression.

## 3 Treatments

Currently, the clinical intervention for patients with SCAs primarily focuses on symptomatic alleviation and functional maintenance because of the absence of effective etiologic treatments, as no medications have been authorized for routine use (Braga Neto et al., 2016). Research on the pathogenesis of SCAs has provided promising therapeutic targets for disease-modifying therapies. This group of diseases may require common therapeutic strategies as well as individualized treatments for the genetic causes of specific SCAs. Since most SCAs are caused by the dominant action of mutant genes, silencing or reducing the expression of the corresponding genes, transcripts, or proteins they encode is a compelling therapeutic strategy. Current therapeutic approaches include gene therapies to reduce toxic gene products, the use of pharmacological molecules to target the affected downstream pathways, and non-pharmacological therapies (Keiser et al., 2016). This review focuses on the latest breakthroughs, challenges, and future directions in gene editing technologies, RNA interference (RNAi), antisense oligonucleotides (ASOs), stem cell technologies, and pharmacologic treatments (Table 3). Each of these therapies has undergone preclinical studies; however, owing to technological bottlenecks, gene editing technologies and RNAi are still in the preclinical stage, whereas others have successively progressed to clinical trials. Early disease intervention is crucial and studies have revealed that some patients with SCAs have a preataxia phase that lasts for several years before the emergence of noticeable ataxia symptoms. During this time, neurons may still be able to be repaired to some extent. Therefore, it is necessary to conduct early preventive trials and develop sensitive biomarkers for patients with SCAs (Maas et al., 2015).

**Table 3:** Key conclusions of some spinocerebellar ataxia models.

SCAs Models	Therapy	Route of administration	Key conclusion	Ref.
SCA1 patients-derived fibroblasts	*ATXN1*-sgRNAs/Cas9	Nucleofection	ATXN1 expression was significantly down-regulated with no major effects on cell viability and few off-target effects	Pappadà et al. (2022)
SCA3-iPSCs	*ATXN3*-sgRNAs/Cas9	Nucleofection	The corrected SCA3-iPSCs differentiated normally into neural stem cells and neurons, which could maintain electrophysiological properties, and the abnormal phenotype of SCA3 neurons was reversed	He et al. (2021)
DRPLA, SCA3, SCA7, and HD patients-derived fibroblasts	shRNA targeting CAG	Lentiviral vectors transduction	CAG-targeted shRNA reduces mutant protein levels without significant off-target effects and preferentially inhibits mutant Huntington protein expression	Kotowska-Zimmer et al. (2020)
SCA1 mouse models	Human ATXN1L, artificial miRNA targeting *ATXN1*	Direct delivery of recombinant AAV vectors to the deep cerebellar nuclei	Mice treated with vectors expressing ATXN1L alone showed changes in gene expression and improved locomotion. Restoration of dysregulated gene expression was greater when vectors expressing both components were used	Carrell et al. (2022)
SCA3 mouse models	siRNA targeting *ATXN3*	Intravenous administration of stable nucleic acid lipid particles	These nanoparticles successfully crossed the blood–brain barrier and reduced mutant ATXN3 levels in the mouse cerebellum, ameliorated motor deficits, and reduced cerebellar-related neuropathology	Conceição et al. (2016)
SCA7 mouse models	ASO targeting *ATXN7* ASO targeting CAG	Intravitreal injection	Ataxin-7 ASO reduced retinal ATXN7 expression and protein aggregation, improved cone and rod photoreceptor function and gene expression, and rescued retinal degeneration, achieving a significant beneficial therapeutic response in symptomatic mice. CAG ASO transiently improves retinal disease phenotype	Niu et al. (2018)
SCA13 mouse models	ASO targeting *Kcnc3*	Intracerebroventricular injection	Kv3.3 ASO reduced Kv3.3 channel expression in mutant mice, decreased TBK1 activation, reversed the reduction in the level of Hax-1, and restored the ability to maintain balance on the rotating rod to the level of wild-type mice	Zhang et al. (2021)
SCA1 mouse models	Human umbilical MSCs	Transplantation into the cerebella	MSCs did not differentiate into neurons or astrocytes in the cerebellum and had the effect of attenuating Purkinje cell loss and cerebellar atrophy, improving deterioration of motor coordination and limb muscle contraction, and promoting the expression of some growth-promoting factors	Tsai et al. (2019)
SCA3 mouse models	Human bone marrow MSCs, human bone marrow MSCs secretome (CM)	Single transplantation of MSCs or injection of CM in different brain regions (cerebellum, striatum/ substantia nigra, and spinal cord)	Single CM administration to the cerebellum or basal ganglia resulted in mild but short-lasting improvements in motor deficits in mice, and no benefit was observed with CM administration to other brain regions or single MSCs transplantation	Correia et al. (2021)
SCA3 mouse models	Calpain inhibitor BDA-410	Oral administration	BDA-410 reduced ATXN3 levels, fragment formation, and aggregation, which served to reduce toxicity to cells, prevent cerebellar cell loss and striatal degeneration, mediate neuroprotection, and alleviate motor deficits	Simões et al. (2014)
SCA3 transgenic zebrafish models	BLD-2736, inhibitor of calpain-1, 2, and 9 and cathepsin K	Submersion in drug dissolved in water	BLD-2736 treatment improved the swimming behavior of SCA3 zebrafish larvae and reduced the presence of insoluble protein aggregates, increased the synthesis of LC3II, a key protein in autophagy, and autophagic activity	Robinson et al. (2021)
SCA3 mouse models	Citalopram	Oral administration	After treatment, mice showed improved motor balance and coordination, reduced brainstem ATXN3 intranuclear inclusions and reactive astrogliosis, and decreased facial neuron loss	Teixeira-Castro et al. (2015)
Fibroblast cell lines from patients with SCA2 or ALS-FTD Mouse models of these diseases	siRNA-STAU1, siRNA-mTOR, and rapamycin Stau1 loss-of-function allele mice	Multiple routes	In disease models, STAU1 and mTOR were overabundant, interacted with each other, and inhibited autophagic flux. In cell models, STAU1 was found to bind mTOR mRNA to promote its translation, and when mTOR activity was inhibited, STAU1 levels were reduced. Reducing Staufen1 levels in mice improved autophagy and reduced cell death	Paul et al. (2023)
SCA3 patient iPSC-derived neuron cells models	TFEB nuclear translocation mediated by umbilical cord blood-derived MSCs	Co-culture approach	MSCs with exosomal vesicles induced TFEB nuclear translocation in neuronal cells after co-culture, which may activate autophagy by regulating the AKT/mTOR and AMPK/mTOR signaling pathways, reduce the level and toxic effects of mutant proteins, and the interaction of mutant proteins with Beclin1	Han et al. (2022)
13 patients with SCA3	Trehalose (100 mg daily)	Oral for 6 months	At 6 months, 61% of patients had improved SARA scores, 8-min walk test scores, and quality of life scores. Patients with younger age of onset, shorter disease duration, and lower SARA scores had better responses to trehalose. Oral trehalose was well tolerated, with the main side effects being bloating and diarrhea	Noorasyikin et al. (2020)
38 patients with SCAs and 17 patients with Friedreich’s ataxia	Riluzole (50 mg, twice daily) or placebo	Oral for 12 months	The proportion of patients with reduced SARA scores at 12 months was significantly higher in the riluzole group (50%) than in the placebo group (11%), and only sporadic mild adverse events were reported	Romano et al. (2015)
45 patients with SCA2	Riluzole (50 mg, twice daily) or placebo	Oral for 12 months	SARA scores were not significantly different between the two groups, and brain MRI did not show significant differences in volume changes in cerebellar or brainstem regions	Coarelli et al. (2022)
SCA1 mouse models	Chlorzoxazone-baclofen	Oral administration	The agent improves Purkinje neuron firing in a dose-responsive manner and ameliorates motor dysfunction originating in the cerebellum without affecting muscle strength	Bushart et al. (2021)
36 patients with SCA3	Low-dose VPA (400 mg, twice daily), high-dose VPA (600 mg, twice daily) or placebo	Oral for 12 weeks	Motor function was significantly improved in the VPA group compared to the placebo group, with a greater decrease in SARA scores in the high-dose group. Common adverse reactions included dizziness and loss of appetite, which occurred primarily in the high-dose group	Lei et al. (2016)
29 patients with SCA2	NeuroEPO (1.0 mg daily, 3 days per week) or placebo	Nasal administration for 6 months	SCAFI and SARA scores improved in the NeuroEPO group, but a high placebo effect was observed, and only one secondary outcome measure, saccade latency, was significantly lower	Rodriguez-Labrada et al. (2022)
9 patients with SCA38	DHA (600 mg daily)	Oral for 2 years	Clinical symptoms of the patients were stable, SARA or ICARS scores improved, and a significant increase in FDG-PET cerebellar metabolism identified oral DHA as an effective treatment with no observed side effects	Manes et al. (2019)

SgRNA, small guide RNA; iPSCs, induced pluripotent stem cells; DRPLA, dentatorubral-pallidoluysian atrophy; SCA, spinocerebellar ataxia; HD, Huntington’s disease; shRNA, short hairpin RNA; miRNA, microRNA; AAV, adeno-associated virus; siRNA, small interfering RNA; ASO, antisense oligonucleotides; MSCs, mesenchymal stem cells; ALS-FTD, amyotrophic lateral sclerosis-frontotemporal dementia; mTOR, mechanistic target of rapamycin; TFEB, transcription factor EB; SARA, Scale for the Assessment and Rating of Ataxia; VPA, valproic acid; SCAFI, spinocerebellar ataxia functional index; DHA, docosahexaenoic acid; FDG-PET, 18-fluorodeoxyglucose-positron emission tomography; ICARS, International Cooperative Ataxia Rating Scale.

### 3.1 Gene editing strategy

**Figure 3:**
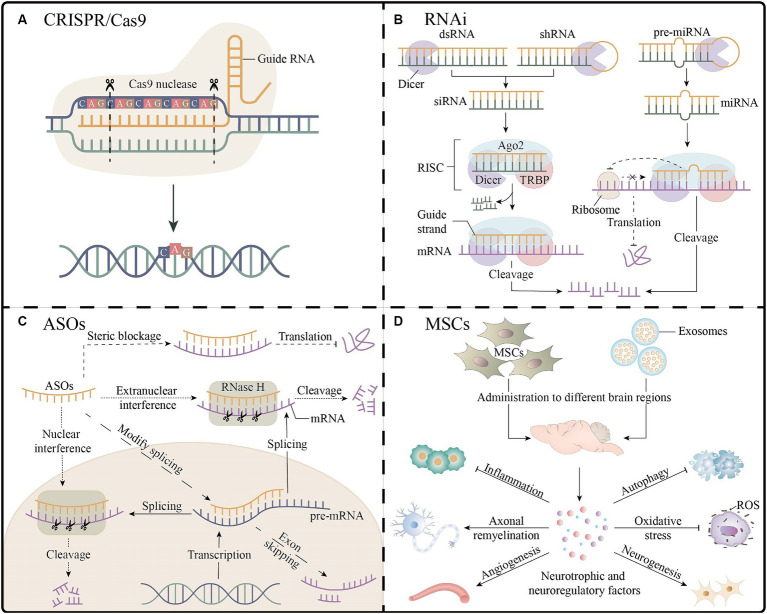
Gene therapy and stem cell therapy for spinocerebellar ataxias. **(A)** The CRISPR/Cas9 platform enables gene editing by guiding RNA strands to bind to target DNA, as shown in the figure for the knockdown of the expanded CAG repeats. **(B)** Three RNAi molecules mediate the process of mRNA cleavage and inhibition of translation outside the nucleus. **(C)** The ASOs inhibit gene expression through mRNA cleavage, interference with splicing, and steric blockage. **(D)** After entering different regions of the brain, MSCs or their exosomes secrete a variety of neuroprotective or neuroregulatory factors, which may have the effects of promoting neurogenesis processes (e.g., neuronal proliferation, differentiation, and myelination), improving angiogenesis, antioxidant, reducing inflammation, and inhibiting apoptosis, which is beneficial to neuronal cell survival and neurological function recovery. Ago2, argonaute2; ASOs, antisense oligonucleotides; CRISPR, clustered regularly interspaced short palindromic repeats; dsRNA, double-stranded RNA; miRNA, microRNA; MSCs, mesenchymal stem cells; RISC, RNA-induced silencing complexes; RNAi, RNA interference; ROS, reactive oxygen species; shRNA, short hairpin RNA; siRNA, small interfering RNA; TRBP, TAR RNA binding proteins.

Gene editing strategies targeting mutant alleles enable precise and permanent gene correction, which can affect all downstream pathways. The main gene editing platform used in the current study was the clustered regularly interspaced short palindromic repeats (CRISPR)/Cas9 system (Figure 3A) (Nouri Nojadeh et al., 2023). A recent study precisely corrected SCA3-iPSCs using a CRISPR/Cas9-mediated homologous recombination strategy, and the corrected iPSCs differentiated normally into neural stem cells and neuronal cells and maintained normal electrophysiological properties. Another study applied the CRISPR/Cas9 approach to SCA1 fibroblasts and revealed that the expression of disease-related proteins was downregulated. These results support the CRISPR/Cas9 system as an effective gene modification technique (He et al., 2021; Pappadà et al., 2022). However, editing strategies have not yet progressed to animal models and clinical trials, and their implementation faces numerous technological challenges, such as optimizing delivery to target cells and minimizing off-target effects.

### 3.2 RNA interference

Targeting mutant mRNA transcripts may be the next best therapeutic alternative. Currently, there are two main oligonucleotide-based therapeutic strategies, namely RNAi and ASOs therapies, that can be applied using either non-allele-specific or allele-specific strategies. Non-allele-specific strategies may target both mutant and wild-type transcripts, resulting in unfavorable side effects by suppressing the expression of wild-type proteins. Allele-specific strategies, which typically focus on mutant alleles through single-nucleotide polymorphisms correlated with the disease allele, are technically challenging but more efficient (Vázquez-Mojena et al., 2021). A better understanding of the cellular function and gene regulation of SCAs proteins through studies in patients and disease models and the development of therapeutic approaches that have minimal impact on wild-type proteins or retain wild-type proteins in normal cellular function while specifically inhibiting mutant proteins will be the future direction of clinical research.

In short, RNAi is a sequence-specific post-transcriptional gene silencing technology activated by double-stranded RNA molecules homologous to target genes, which can be mediated by three functionally different molecules: microRNA (miRNA), small interfering RNA (siRNA) and short hairpin RNA (shRNA); the single-stranded RNA formed after processing with Dicer nucleic acid endonuclease can be bound to RNA-induced silencing complexes to achieve the degradation or translational inhibition of target mRNA (Figure 3B) (Santos et al., 2023). Targeting CAG repeat expansion by shRNA was applied to DRPLA, SCA3, SCA7, and HD patient-derived fibroblasts; a reduction in the expression levels of the mutant proteins was reported to varying degrees without significant off-target effects (Kotowska-Zimmer et al., 2020). The primary mechanism of SCA1 is the toxic gain-of-function of mutant expansion-containing ATXN1, accompanied by a partial loss-of-function in the wild-type. A study combining artificial miRNA targeting ATXN1 and ATXN1 homolog ATXN1L into recombinant adeno-associated virus (AAV) vectors demonstrated that mice that administered the two-component AAV vector showed normalization of gene expression and improvement in motor function, with a better therapeutic effect than that in the mice treated with vectors expressing ATXN1L only (Carrell et al., 2022). A study using non-viral, stable nucleic acid-lipid particles as a vehicle, combined with a short peptide derived from the rabies virus glycoprotein and encapsulating siRNA targeting mutant ATXN3, effectively silenced the expression of mutant ATXN3 in SCA3 mouse models by intravenous administration, leading to a reduction in neuropathological and motor behavioral deficits; this is the first preclinical study examining the benefit of non-invasive systemic administration in polyQ diseases (Conceição et al., 2016). Additionally, RNAi-based therapies have been extensively studied preclinically in SCAs models. This provides a theoretical basis for clinical trials. However, their clinical application faces problems such as poor *in vivo* stability, effectiveness of delivery systems, and off-target effects.

### 3.3 Antisense oligonucleotides

In general, ASOs are single-stranded synthetic antisense oligonucleotides that are complementary to specific mRNA. When combined with the target mRNA, they may cause various mechanisms for blocking gene expression, including RNase H endonuclease-mediated mRNA cleavage, interference with splicing to achieve exon skipping, and interference with translation through steric blockage (Figure 3C) (Vázquez-Mojena et al., 2021). Preclinical studies of ASOs for the treatment of SCAs have yielded promising results, including those on cellular or animal models of SCA1, 2, 3, 7, and 13. A clinical trial of intrathecal injection of non-allele-specific ASOs in patients with SCA3 was initiated in 2022 (NCT05160558) (Coarelli et al., 2023b). Moreover, ASOs are considered the most promising gene therapies for treating SCAs; however, they have shown inconsistent results in clinical studies on patients with other neurodegenerative diseases. Nusersen, a modified ASO, has been approved for intrathecal injection in pediatric and adult patients with spinal muscular atrophy in the United States because of its effectiveness. However, three clinical trials using ASOs to treat patients with HD were interrupted for several reasons, such as worsening of the patients’ clinical rating scales, increased frequency of severe adverse events, and failure of reducing the mutant protein levels in cerebrospinal fluid. Therefore, clinical trials involving patients with SCA3 have received considerable attention (Hoy, 2017; Tabrizi et al., 2022). Intravitreal injection of ASOs targeting ATXN7 mRNA in SCA7 mouse models showed better long-term improvements than those observed with the intravitreal injection of ASOs targeting CAG repeat expansion, such as a significant reduction in ATXN7 expression and aggregation, amelioration of vision loss, and alleviation of retinal histopathology (Niu et al., 2018). Lateral ventricular injection of ASOs targeting Kv3.3 channels has little effect on wild-type mice; however, it suppresses the mRNA and protein levels of the Kv3.3 channel in mouse models of SCA13 and restores the behavior of mutant mice to that of age-matched wild-type mice, suggesting that targeting Kv3.3 expression may be a viable therapeutic approach for treating SCA13 (Zhang et al., 2021). The non-permeability of the blood–brain barrier, invasive delivery methods, and the requirement for multiple repeat administrations impede the clinical application of ASO therapies. The development of less invasive brain delivery methods remains challenging, with AAV vectors, nanoparticles, cell-penetrating peptides, or liposome-mediated delivery being promising alternatives (Lee L. K. C. et al., 2021).

### 3.4 Stem cell therapy

Stem cell technology offers new directions for the treatment of neurodegenerative diseases, possibly via cell replacement or neuronal trophic support. Mesenchymal stem cells (MSCs), with their high availability and paracrine and immunosuppressive effects, are the most widely used cell types for studying SCAs (Figure 3D) (Correia et al., 2023). Transplantation of human umbilical MSCs into the bilateral cerebellar cortex of SCA1 mouse models significantly improve motor behavior, effectively attenuate cerebellar atrophy, and reverse Purkinje cell death (Tsai et al., 2019). A study of single human bone marrow MSCs transplantation or single MSCs secretome administration in relevant brain regions of SCA3 mouse models revealed that MSCs secretome administration was more beneficial, especially when administered to the cerebellum and basal ganglia. However, the therapeutic effect was mild and transient, raising concerns about the replacement of MSCs by MSCs by-products, the efficacy and risk of repeated systemic administration, minimal invasiveness, and effective routes of administration (Correia et al., 2021). There are ongoing clinical studies related to MSCs that involve patients with SCA1, 2, 3, and 6, which have shown varying degrees of improvement in symptoms and ataxia scale scores. However, a systematic evaluation and meta-analysis based on previous clinical trials showed low and statistically indistinguishable evidence supporting functional recovery in patients with SCAs, which could possibly be attributed to some limitations such as the few conducted clinical studies, limited sample sizes, and low-quality study designs (Appelt et al., 2021). Therefore, large-scale, high-quality clinical trials are needed to determine the long-term efficacy, tolerability, and safety of stem cell therapies, and explore the optimal therapeutic doses, frequency, and routes of administration.

### 3.5 Pharmacological therapy

#### 3.5.1 Reduction of toxic protein levels

Therapies targeting downstream toxic effects should primarily aim at reducing toxic protein levels, including inhibiting the production of toxic protein fragments, reducing mutant protein aggregation, and inducing autophagy to stimulate protein clearance (Buijsen et al., 2019). One approach to inhibit the production of toxic fragments is to inhibit the cleavage of mutant proteins by proteolytic enzymes. One study found that calpain-mediated expression of ataxin-3 cleavage fragments may induce mitochondrial fragmentation and cristae alterations, leading to a significant reduction in mitochondrial respiratory capacity, affecting proper clearance of damaged mitochondria by interfering with mitophagy, and increasing susceptibility to apoptosis (Harmuth et al., 2018). Oral administration of the calpain inhibitor BDA-410 to the SCA3 mouse model reduced the toxic fragments of the mutant ATXN3 protein, decreased the number of its inclusions, and prevented cerebellar cell loss, suggesting that calpain inhibitors may mediate neuroprotection and inhibit proteolysis (Simões et al., 2014). One study tested the efficacy of a novel small-molecule calpain inhibitor compound, BLD-2736, in the SCA3 transgenic zebrafish model and found that the animals showed a dose-dependent improvement in motor behavior, a reduction in insoluble protein aggregates, and an increase in the expression of key proteins for autophagy (Robinson et al., 2021). In the HD mouse model, calpain inhibition had a protective effect, increasing autophagosome levels, improving motor signs, and delaying the onset of tremor, and prolonged calpain inhibition did not lead to any significant deleterious phenotypes (Menzies et al., 2015). Another study found that inhibition of calpains, which is prevalent in the heart after ischemia–reperfusion, improved mitophagy in cardiac cells through processes such as increased translocation of LC3B to mitochondria (Chen et al., 2019). Thus, these studies suggest that calpain inhibitors may have a dual protective effect of reducing cleavage of ATXN3 and stimulating mitophagy or general autophagy. However, for most polyQ diseases, the proteolytic enzymes responsible for the cleavage of their mutant proteins have not been identified, and the inhibition of proteolytic enzymes may affect a wider range of proteins beyond mutant polyQ proteins, leading to side effects. Therefore, the feasibility of this strategy is limited. Citalopram was reported to inhibit ATXN3 aggregation and neurotoxicity; when tested in a mouse model of SCA3, it improved motor and coordination functions, reduced the number of intranuclear inclusions in the brainstem, and rescued motor neuron loss. Moreover, it may affect the folding and stabilization of ATXN3, thereby inhibiting its aggregation, rather than removing the mutant protein. Thus, citalopram may be a viable option to halt the SCA3 progression. Further studies are needed to validate its applicability in other patients or SCA-related diseases (Teixeira-Castro et al., 2015).

Impaired autophagy occurs in a variety of neurodegenerative diseases. Therefore, the activation of autophagy may be a viable approach for treating neurodegeneration, and accordingly, multiple pathways targeting autophagy based on genes, stem cells, and pharmacology have been investigated. The mechanistic target of rapamycin (mTOR) kinase is a key regulator of different steps in the autophagy process, and its activity is regulated by complex interactions between upstream regulators, including AMPK, PI3K/AKT pathway, and ERK1/2 (Lee J. H. et al., 2021). A recent study revealed that the stress granule protein STAU1 is overexpressed in multiple models of neurodegenerative diseases and may interact with mTOR, leading to the overactivation of mTOR and inhibition of autophagic flux. Normalization of mTOR activity and some autophagy marker proteins was shown in an siRNA-STAU1 cell model and a mouse model with a Stau1 loss-of-function allele, suggesting that STAU1 might be a novel target for regulating autophagy (Paul et al., 2023). The transcription factor EB (TFEB) regulates the expression of key genes for lysosomal biogenesis and autophagy at the transcriptional level. The mTOR can phosphorylate TFEB and anchor it to the cytoplasm; whereas in the mTOR-inactivated state, TFEB translocates to the nucleus, thereby promoting transcription (Menzies et al., 2017). A study that co-cultured umbilical cord blood-derived MSCs with iPSC-derived neurons from patients with SCA3 showed that MSCs induced TFEB nuclear translocation in neuronal cells, activated autophagy, and reduced the intracellular levels of mutant proteins by modulating the AKT/mTOR and AMPK/mTOR signaling pathways (Han et al., 2022). Trehalose is a widely used mTOR-independent autophagy inducer in neurodegenerative models, and clinical trials of oral trehalose have been conducted in patients with SCA3. Clinical symptoms showed improvements or were delayed in most patients; patients with younger age of onset, shorter disease duration, and lower Scale for the Assessment and Rating of Ataxia (SARA) scores displayed better responses to trehalose (Noorasyikin et al., 2020). Other pharmacological inducers, such as lithium, temsirolimus, cordycepin, carbamazepine, and n-butylidenephthalide, were reported to enhance autophagy in preclinical studies related to SCA1, SCA2, and SCA3, with cellular or animal models exhibiting decreased levels of mutant proteins, reduced number of aggregates, and improved neuropathology (Paulino and Nóbrega, 2023). Autophagy is currently one of the most studied targets in neurodegenerative diseases. The continued emergence of novel autophagy-inducing approaches suggests broad prospects for the development of this strategy, which may be a promising treatment alternative for patients with SCAs.

#### 3.5.2 Improvement of ion channel dysfunction

Pharmacological agents potentially target impaired downstream mechanisms by modulating disturbances in cerebellar electrophysiological circuits, correcting transcriptional dysregulation, improving mitochondrial dysfunction, and reducing other factors that lead to neuronal degeneration and damage. Riluzole is an anti-glutamatergic compound that may exert neuroprotective effects by inhibiting glutamatergic signaling-induced toxicity and opening calcium-activated potassium channels to modulate cerebellar neuronal firing. A randomized double-blind controlled trial included 55 patients diagnosed with SCAs and Friedreich ataxia who were divided into an oral riluzole group and a placebo group. After 12 months, the proportion of patients with decreased SARA scores was significantly higher in the oral riluzole group than in the placebo group. Furthermore, in a systematic review of pharmacological agents for SCAs, riluzole received a grade A recommendation for the treatment of ataxia symptoms (Romano et al., 2015; Yap et al., 2022). However, a homogeneous clinical trial of riluzole for the treatment of patients with SCA2 that was conducted in France reported less favorable outcomes, with no significant improvement in the imaging changes or SARA scores of the patients (Coarelli et al., 2022). Thus, riluzole may have different effects on different clinical forms of ataxia, and its pharmacological effects need to be evaluated in various populations with specific SCAs. Chlorzoxazone and baclofen are potassium channel-activating compounds that may target BK channels, which are large conductance calcium-activated potassium channels, and their reduced expression has been reported in SCA1, SCA2, and SCA7 mouse models. Reduced expression of BK and CaV3.1 ions in the cerebellum of SCA1 mice was also demonstrated in a recent study which combined the application of chlorzoxazone and baclofen with a resulting improvement in the physiology of Purkinje neuron and cerebellar motor dysfunction in mice and identification of the dose level to minimize cerebellar extracerebellar toxicity for potentially applying it to future clinical trials (Bushart et al., 2018, 2021). Given that changes in Purkinje neuronal excitability are present in various SCAs, ion channel modulators, particularly molecules with high target specificity, may be attractive approaches for symptomatic improvement.

#### 3.5.3 Correction of transcription dysregulation

Transcriptional dysregulation is an important pathological process in polyQ diseases, and elevated histone acetylation levels favor transcriptional activation (Xiang et al., 2018). Several cellular and animal models of SCAs have demonstrated that some histone deacetylase inhibitors, such as sodium butyrate, trichostatin A, and valproic acid (VPA), can rescue histone hypoacetylation and transcriptional defects (Buijsen et al., 2019). A previous study determined the maximum tolerated single-dose VPA in patients with SCA3 to be 800 mg twice daily and subsequently tested the efficacy of long-term oral VPA in 36 patients with SCA3. After 12 weeks, patients in the low-dose (400 mg, twice daily) and high-dose (600 mg, twice daily) groups showed significant improvements in their motor function compared with that for the placebo group, with a more significant decrease in the SARA scores in the high-dose group. Based on the observed results, the combination of riluzole and VPA could potentially result in greater therapeutic efficacy (Lei et al., 2016). However, further studies are needed to determine the extent to which transcriptional dysregulation is involved in the pathogenesis of various SCAs and determine whether targeting this pathway will yield the desired outcomes.

#### 3.5.4 Improvement of mitochondrial dysfunction

Many experiments have suggested that erythropoietin (EPO) may have neurotrophic effects, and the non-hematopoietic EPO analog, NeuroEPO, attenuated the glutamate excitotoxicity-induced oxidative stress, neuronal apoptosis, and neuroinflammation by maintaining mitochondrial membrane integrity, upregulating Bcl-2, and inhibiting Bax, cytochrome C, and caspase-3 (Garzón et al., 2018). A randomized, double-blind, placebo-controlled trial using the nasal administration of NeuroEPO in 34 patients with SCA2 was conducted to explore its potential efficacy. It showed a significant improvement in the saccade latency secondary outcome metric only. This, provides evidence of its feasibility for future clinical trials (Rodriguez-Labrada et al., 2022).

#### 3.5.5 Other pharmacological molecules

The ELOVL5 mutation in patients with SCA38 leads to abnormalities and mislocalization of the encoded protein, causing reduced serum levels of the end-product, docosahexaenoic acid (DHA). A study demonstrated that the long-term oral DHA administration to patients with SCA38 slowed the onset and progression of symptoms and significantly improved cerebellar metabolism; accordingly, oral DHA (600 mg/day) administration is an effective treatment for SCA38 and it is essential to develop therapeutic approaches to treat specific SCA subtypes.

Rovatirelin, amantadine, buspirone, and varenicline are other agents that were suggested in previous studies to potentially alleviate symptoms in patients with SCAs. However, clinical trials investigating these drugs have not shown significant effects (Ghanekar et al., 2022). In summary, randomized controlled trials of pharmacological agents for the treatment of SCAs have been extensively conducted worldwide; however, there is no clear evidence of their potential benefits. It is important to note that most of these drugs are pleiotropic, such as the antidepressant effect of citalopram, the central muscle relaxant effect of chlorzoxazone-baclofen, and the anticonvulsant effect of valproic acid. Their possible short-and long-term side effects in patients with SCAs need to be determined in further studies. In addition, the potential applications of these pharmacological agents need to be validated in future clinical trials with adequate sample sizes, rigorous preclinical studies, and more comprehensive data analyses.

## 4 Discussion

The development of therapeutic approaches for SCAs is inextricably linked to studying their pathogenesis. For toxic gain-of-function mutations, various sites of the pathogenic cascade should be inhibited to reduce the expression of the mutant protein, whereas for loss-of-function mutations, attempts should be made to restore the defective protein to its baseline levels. Gene therapy suggests the possibility of rectifying the underlying factors of SCAs. Many preclinical studies have reported varying degrees of the inhibition of target gene expression *in vivo*, but its clinical application still faces difficulties such as the development of strategies to specifically target the mutated genes, selection of appropriate vectors and delivery modalities, potential toxicity risks, and ethical challenges for patient application. Stem cell therapies primarily focus on replacing degenerated and damaged neural cells; while key issues such as the route of administration, dosage, source, and culture conditions still need to be addressed. Other therapeutic strategies and pharmacological molecules are being developed rapidly and show promise in improving clinical symptoms and slowing disease progression. Targeting autophagy is a meaningful therapeutic strategy, as its upregulation enhances the clearance of major pathogenic toxicants, may have additional protective effects, and may be beneficial in patients with a wide range of neurodegenerative diseases. Cerebellar circuit perturbations and motor dysfunctions caused by Purkinje cell electrophysiological dysfunction, a common feature of many SCAs, involve changes in a variety of ion channels; thus, approaches to target ion channels with higher specificity and potency should be designed in the future (Bushart et al., 2018). Elevated levels of oxidative stress, mitochondrial dysfunction, and activation of intrinsic apoptotic pathways play crucial roles in cell death in neurodegenerative diseases and provide a theoretical rationale for the use of antioxidants to maintain mitochondrial integrity, modulate redox status, and attenuate neurotoxicity (Garzón et al., 2018). It is worth noting that some SCAs mutant proteins may affect multiple cellular processes, and determining the extent to which each process is involved in the pathogenesis, targeting important pathways, and implementing combination therapies targeting multiple pathways may yield desirable therapeutic outcomes.

The ongoing development and application of genetic testing techniques, such as next-generation sequencing, long-read sequencing, and bioinformatics analyses have identified new mutant genes or new variant forms associated with SCAs. They have helped in improving the diagnostic accuracy of SCAs and discovering novel disease mechanisms. Future studies on the cellular functions of the causative proteins of various SCAs subtypes should be conducted to design novel and specific therapeutic approaches. The development of effective treatments for SCAs poses a significant challenge owing to the heterogeneity of its pathogenesis and pathological changes, the fact that each subtype may require specific treatment, and the insufficient sample size available for clinical trials of SCAs owing to its rare incidence. Previous therapeutic studies have favored common SCAs and provided some promising avenues that suggest the possible success of disease-modifying therapies for common SCAs. Animal and human trials suggest that initiating treatment in the early or pre-onset phase of the disease often results in better therapeutic outcomes, whereas in the most severe cases, it may not be very effective, requiring early diagnosis and intervention. In addition, the selection of appropriate outcome metrics and biomarkers in clinical trials is essential for understanding the underlying mechanisms and targets involved in each treatment, monitoring disease progression in different SCA genotypes, and assessing treatment efficacy. Therefore, the development of relevant and novel biomarkers is necessary to refine clinical trial design; moreover, high-quality clinical trials are needed to assess the efficacy and safety of the various treatments for SCAs.

## Author contributions

Z-TC: Writing – original draft. Z-TM: Writing – original draft. RY: Writing – original draft. J-JL: Writing – original draft. S-SJ: Writing – original draft. J-LZ: Writing – original draft. F-TZ: Writing – original draft. PY: Writing – review & editing. MD: Writing – review & editing.
